# Anticancer Effects of Emodin on HepG2 Cell: Evidence from Bioinformatic Analysis

**DOI:** 10.1155/2019/3065818

**Published:** 2019-05-19

**Authors:** Rui-sheng Zhou, Xiong-Wen Wang, Qin-feng Sun, Zeng Jie Ye, Jian-wei Liu, Dai-Han Zhou, Ying Tang

**Affiliations:** ^1^The First Affiliated Hospital of Guangzhou University of Chinese Medicine, Guangzhou University of Chinese Medicine, Guangzhou, China; ^2^Guangzhou University of Chinese Medicine, Guangzhou, China; ^3^Stomatological Hospital of Shandong University, Shandong, China; ^4^Jinan Stomatological Hospital, Shandong, China; ^5^Lingnan Medical Research Center of Guangzhou University of Chinese Medicine, Guangzhou, China

## Abstract

Hepatocellular carcinoma (HCC) is a primary cause of cancer-related death in the world. Despite the fact that there are many methods to treat HCC, the 5-year survival rate of HCC is still at a low level. Emodin can inhibit the growth of HCC cells in* vitro* and in* vivo*. However, the gene regulation of emodin in HCC has not been well studied. In our research, RNA sequencing technology was used to identify the differentially expressed genes (DEGs) in HepG2 cells induced by emodin. A total of 859 DEGs were identified, including 712 downregulated genes and 147 upregulated genes in HepG2 cells treated with emodin. We used DAVID for function and pathway enrichment analysis. The protein-protein interaction (PPI) network was constructed using STRING, and Cytoscape was used for module analysis. The enriched functions and pathways of the DEGs include positive regulation of apoptotic process, structural molecule activity and lipopolysaccharide binding, protein digestion and absorption, ECM-receptor interaction, complement and coagulation cascades, and MAPK signaling pathway. 25 hub genes were identified and pathway analysis revealed that these genes were mainly enriched in neuropeptide signaling pathway, inflammatory response, and positive regulation of cytosolic calcium ion concentration. Survival analysis showed that LPAR6, C5, SSTR5, GPR68, and P2RY4 may be involved in the molecular mechanisms of emodin therapy for HCC. A quantitative real-time PCR (qRT-PCR) assay showed that the mRNA levels of LPAR6, C5, SSTR5, GPR68, and P2RY4 were significantly decreased in HepG2 cells treated with emodin. In conclusion, the identified DEGs and hub genes in the present study provide new clues for further researches on the molecular mechanisms of emodin.

## 1. Introduction

Hepatocellular carcinoma (HCC) is a severe disease of the digestive system and is the sixth most common and second leading cause of death due to cancer. While the early stage of HCC can be successfully treated by surgical resection and liver transplantation, most HCC cases are diagnosed only at the late stage, when treatment options are more limited [1–3]. Targeted therapies using sorafenib, regorafenib, and lenvatinib, as well as immunotherapy with a PD-1 inhibitor (nivolumab), have been approved for the treatment of advanced-stage HCC [4–7]. However, there remains an urgent need for the identification and development of novel therapeutic agents and strategies for the treatment of HCC.

**Figure 1:**
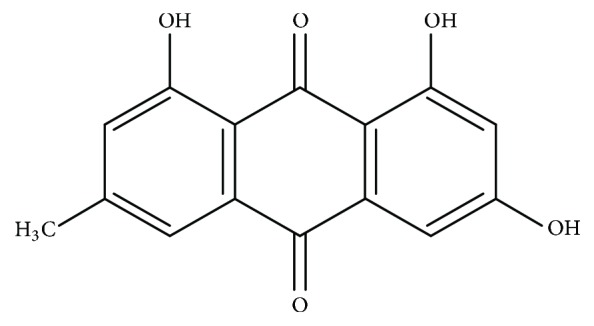
Chemical structure of emodin.

Emodin (1,3,8-trihydroxy-6-methylanthraquinone; Figure 1) is an active ingredient derived from* Polygonum cuspidatum* [8],* Rheum palmatum* [9],* Cassia occidentalis* [10], and P*olygonum multiflorum* [11] and has been used in China for many centuries. The role of emodin as an anticancer drug has been previously described. Modern pharmacological studies have revealed that emodin exhibits various biological activities, such as apoptosis-inducing and antiproliferative effects in breast cancer [12], pancreatic cancer [13], prostate cancer [14], gastric cancer [15], lung carcinoma [16], colon cancer [17], and HCC [18]. Furthermore, emodin can inhibit metastasis, invasion, and migration in HCC and breast cancer [19, 20]. Dong* et al.* showed that emodin induces apoptosis in human HCC [21]. Hsu* et al.* confirmed that emodin inhibited the growth of hepatoma cells [22]. Numerous studies have confirmed the therapeutic effect of emodin on liver cancer. Thus, it is necessary to identify the key genes associated with emodin in HepG2 cells by conducting comprehensive bioinformatic analysis.

High-throughput technologies, such as transcriptome, protein, metabolite, and RNA sequencing, are high accuracy tools that can be used to identify biomarkers for the treatment, diagnosis, and prognosis of various diseases [23]. RNA sequencing (RNA-seq) uses deep-sequencing technologies to provide precise information regarding transcription profiles. The use of RNA-seq in analyzing the effects of drug treatments presents significant advantages including the identification of differentially expressed genes (DEGs) associated with the drug. Network and functional enrichment analyses are also beneficial in understanding the molecular mechanisms underlying drug action.

Although emodin exhibits good clinical efficacy, its gene regulatory mechanisms in liver cancer cells have not been systematically elucidated. Therefore, it is necessary to measure the expression levels of DEGs in cancer cells after treatment with emodin and to systematically analyze the functions of these genes. To overcome the aforementioned issue, the transcriptomes of emodin-treated HepG2 cells were profiled using RNA-seq method. The DEGs induced by emodin treatment were then examined in more detail using a series of analysis tools. The hub genes were extracted, and their corresponding expression levels were compared. A series of survival analyses was then conducted to determine whether the hub genes are correlated with poor prognosis. The relationship between the hub genes and tumor progression in patients with HCC was analyzed. Finally, statistical analysis of functional DEGs was performed, and their potential possible contributions to the anticancer effects of emodin were discussed.

## 2. Materials and Methods

### 2.1. Drug

Emodin was purchased from the Chinese Medicine Center in Beijing, dissolved in dimethylsulfoxide (DMSO) at a concentration of 100 mM, and stored at −20°C. The compound was diluted in the appropriate medium to 25, 50, 75, and 100 *μ*M immediately before use. The final concentration of DMSO was <0.1%.

### 2.2. Cell Culture

Human hepatocellular carcinoma HepG2 cells were purchased from cell bank of the institute of Biochemistry and Cell Biology, Shanghai Institutes for Biological Sciences, Chinese Academy of Science (http://www.cellbank.org.cn/). Cells were cultured in RPMI1640 medium (GIBCO, Grand Island, NY, USA), with 10% fetal bovine serum (FBS, Gemini, US), 100 U/ml penicillin, and 100 mg/ml streptomycin in a humidified atmosphere with 5% CO2 at 37°C (Thermo Fisher Science, MA, USA). The cells with 80% confluence were treated by emodin of different concentrations. In this study, we have pooled 3 biological repeats into one RNA-seq experiment to achieve the same amount of total RNA content. More specifically, at the cell culturing section, cancer cells in 6 different petri dishes were divided into two equal groups. One group was used as emodin treated group, and the other was used as control/untreated group.

### 2.3. Cell Proliferation Assay

Cell Counting Kit-8 assay was used to measure cell proliferation. HepG2 cells were seeded in 96-well plates at 5 × 10^3^ cells/well and incubated at 37°C in complete medium for 24 h before being treated with increasing concentrations of emodin for up to 72 h. Then the cells were incubated with 10 *μ*l CCK8 at 37°C for 2 h. Absorbance at 450 nm was determined using a microplate reader as recommended by the manufacturer. All experiments were performed in triplicate and repeated at least three times with essentially similar results. The inhibition rate was calculated as follows: inhibition rate (%) = [average OD value (control)-average OD value (medication)]/average OD value (control) × 100%. The IC50 value was calculated on the nonlinear regression fit method by the GraphPad Prism software.

### 2.4. RNA-Seq and Analysis

We have pooled 3 biological repeats into one RNA-seq experiment to achieve the same amount of total RNA content. More specifically, at the cell culturing section, cancer cells in 6 different petri dishes were divided into two equal groups. One group was used as emodin treated group, and the other was used as control/untreated group. Briefly, after being treated with emodin for 72 h, total RNA was extracted using Trizol reagent (Invitrogen, Carlsbad, CA, USA). We use Agilent 2100 Bio analyzer (Agilent RNA 6000 Nano Kit) to do the total RNA sample QC: RNA concentration, RIN value, 28S/18S, and the fragment length distribution. We filter the low quality reads (more than 20% of the bases qualities are lower than 10), reads with adaptors, and reads with unknown bases (N bases more than 5%) to get the clean reads. Then we assembled those clean reads into Unigenes, followed with Unigene functional annotation, SSR detection and calculate the Unigene expression levels and SNPs of each sample. Finally, we identify DEGs (differential expressed genes) between samples and do clustering analysis and functional annotations. We use internal software SOAPnuke to filter reads, as follows: (1) remove reads with adaptors; (2) remove reads in which unknown bases (N) are more than 10%; (3) remove low quality reads (we define the low quality read as the percentage of base whose quality is lesser than 15 and is greater than 50% in a read). After filtering, the remaining reads are called “Clean Reads” and stored in FASTQ format. We use HISAT (Hierarchical Indexing for Spliced Alignment of Transcripts) to do the mapping step because HISAT is much faster and sensitive and is a high accuracy analysis software [24]. We mapped clean reads to reference using Bowtie2 [25], and then calculated gene expression level with RSEM [26]. RSEM is a software package for estimating gene and isoform expression levels from RNA-Seq data. Then, we calculate Pearson correlation between all samples using cor, perform hierarchical clustering between all samples using hclust, perform PCA analysis with all samples using princomp, and draw the diagrams with ggplot2 with fuctions of R. We detect DEGs with DEGseq [27]. DEGs at each stage or site were used for further analyses of GO (gene ontology) Molecular Function and KEGG pathways using by the Database for Annotation, Visualization, and Integrated Discovery (DAVID: http://david.abcc.ncifcrf.gov).

### 2.5. PPI Network Construction and Module Analysis

The PPI network was predicted using Search Tool for the Retrieval of Interacting Genes (STRING; http://string-db.org) [28] online database. Analyzing the functional interactions between proteins may provide insights into the mechanisms of generation or development of diseases. In the present study, PPI network of DEGs was constructed using STRING database, and an interaction with a combined score >0.7 was considered statistically significant. Cytoscape is an open source bioinformatics software platform for visualizing molecular interaction networks [29]. The plug-in ClusterONE (version 1.0) of Cytoscape is an APP for clustering a given network based on protein-protein interaction networks [30]. The PPI networks were drawn using Cytoscape and the most significant module in the PPI networks was identified using ClusterONE. Subsequently, the KEGG and GO analyses for genes in this module were performed using DAVID.

### 2.6. Hub Genes Selection and Analysis

The hub genes were selected with degrees ≥10 [31]. A network of the genes and their coexpression genes was analyzed using cBioPortal (http://www.cbioportal.org) online platform [4, 14]. The overall survival and disease-free survival analyses of hub genes were performed using Kaplan-Meier curve in cBioPortal [32].

### 2.7. Quantitative Real-Time PCR

A quantitative real-time PCR (qRT-PCR) assay was developed for the detection and quantification of AXIN2, WNT5B, WNT3A, CATENIN, and GSK3B transcripts using ACTIN as an endogenous control. The primers used in this study were designed as follows: ACTIN forward 5′-CACCCAGCACAATGAAGATCAAGAT-3′; reverse 5′-CCAGTTTTTAAATCCTGAGTCAAGC-3′. P2RY4 forward 5′-CTGGACTGTTGGTTTGATGAGGA-3′; reverse 5′-CAGCGACAGCACATACAAGGT-3′; C5 forward 5′-GGAGTGACGGTGCTGGAGTTT-3′; reverse 5′-CCCTCGTGCCAAAGTGGATAA-3′; SSTR5 forward5′- CTACATTCTCAACCTGGCAGTGG-3′; reverse 5′-GCTCATGACTGTCAGGCAGAAGA-3′. LPAR6 forward 5′-GGTAAGCGTTAACAGCTCCCAC-3′; reverse 5′- CATTTCGGACTTTGAGGACGC-3′. GPR68 forward 5′-CAACTCCTCGATGAGCTGTACCA-3′; reverse 5′- AGGTAGCCGAAGTAGAGGGACA-3′. qRT-PCR was performed in a 20 *μ*L mixture containing 2 *μ*L of the cDNA preparation, 10 *μ*L 2x SYBR Green Premix ExTaq (Takara), and 10 *μ*M primer on an ABI 7500 Real-Time PCR System (Applied Biosystems, Grand Island, NY, USA). The PCR conditions were as follows:10 min at 95°C, followed by 40 cycles of 15s at 95°C, and 1 min at 60°C. Each sample was tested in triplicate. Threshold values were determined for each sample/primer pair, and the average and standard errors were calculated.

## 3. Results

### 3.1. Emodin Inhibits HepG2 Cell Growth in the Time- and Dose-Dependent Manner

**Figure 2:**
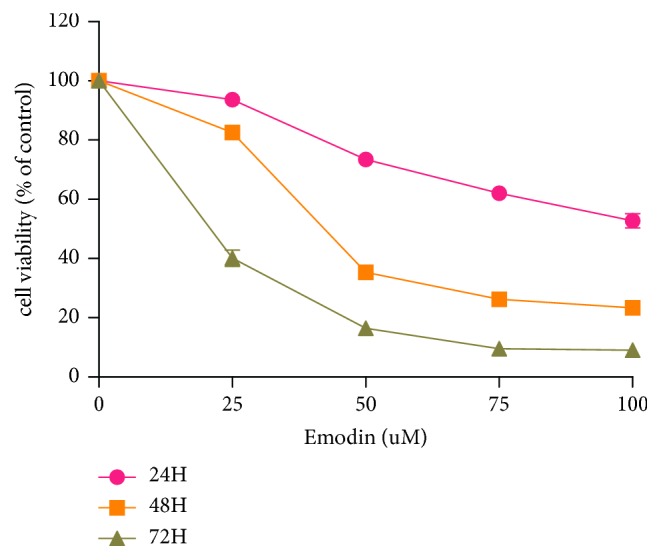
Emodin inhibits HepG2 cell growth in the time- and dose-dependent manner. HepG2 cells were treated with increased concentrations of emodin for up to 72 hrs to examine the cell viability. The cell viability was determined using the CCK8 assay as described in Materials and Methods and was expressed as percentage of control in the mean ± SD of three separate experiments.

We analyzed the effect of emodin on HepG2 cell proliferation. CCK8 assays were performed using the HCC cell line, HepG2, after treatment with different concentrations of emodin for 24, 48, and 72 h. As shown in Figure 2, emodin decreased cell viability in a dose- and time-dependent manner, and the 50% inhibitory concentration (IC_50_) observed at 72 h was 19.12 *μ*M in HepG2 cells.

### 3.2. RNA-Seq Landscape

Treatment of HepG2 cells via emodin dose-response assays revealed that emodin had IC_50_ values of about 20 *μ*M at 72 h. The whole transcriptomic profiles of HepG2 cells and of those treated with emodin were assessed at base-pair resolution via RNA sequencing. After removing the low-quality reads, the raw reads from each sample were mapped to a reference genome (Table 1).

### 3.3. Identification of DEGs

**Figure 3:**
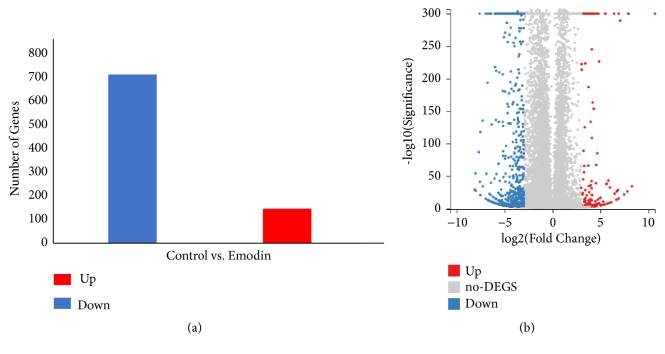
Column diagram and Volcano Plot of DEGs. DEGs were selected with a log⁡2(fold change) >3 and adj. P-value <0.001 among the mRNA expression of emodin and control. (a) Column diagram of DEGs. (b) Volcano Plot of DEGs. Upregulated genes are marked in light red; downregulated genes are marked in light blue.

DEGs were screened and generated by comparing the emodin samples to the control samples. As shown in the column diagram (Figure 3(a)) and volcano plot (Figure 3(b)), the analysis identified 859 DEGs comprising 712 downregulated genes and 147 upregulated genes that satisfy the criteria log⁡2(fold change) >3 and adj. p-value <0.001.

### 3.4. KEGG and GO Enrichment Analyses of DEGs

To determine the biological classifications of the DEGs, functional and pathway enrichment analyses were performed using DAVID, and the GO functional enrichments of the upregulated and downregulated genes were determined at a p-value of less than 0.05. GO analysis results showed that changes in biological processes (BP) of DEGs were significantly enriched in cellular heat acclimation, positive regulation of apoptotic process, response to lipopolysaccharide, negative regulation of inclusion body assembly, negative regulation of extrinsic apoptotic signaling pathway in absence of ligand, negative regulation of endopeptidase activity, extracellular matrix organization, homophilic cell adhesion via plasma membrane adhesion molecules, wound healing, and fibrinolysis (Table 2). Changes in cell component (CC) of DEGs were mainly enriched in extracellular space, extracellular region, proteinaceous extracellular matrix, integral component of membrane, integral component of plasma membrane, and myofibril (Table 2). Changes in molecular function (MF) were mainly enriched in ATPase activity, coupled, serine-type endopeptidase activity, serine-type peptidase activity, bradykinin receptor activity, protein domain specific binding, calcium ion binding, serine-type endopeptidase inhibitor activity, extracellular matrix structural constituent, structural molecule activity, and lipopolysaccharide binding (Table 2). KEGG pathway analysis revealed that the DEGs were mainly enriched in complement and coagulation cascades, amoebiasis, ECM-receptor interaction, protein digestion and absorption, platelet activation, MAPK signaling pathway, rheumatoid arthritis, estrogen signaling pathway, legionellosis, and metabolism of xenobiotics by cytochrome P450 (Table 2).

### 3.5. PPI Network Construction and Module Analysis

**Figure 4:**
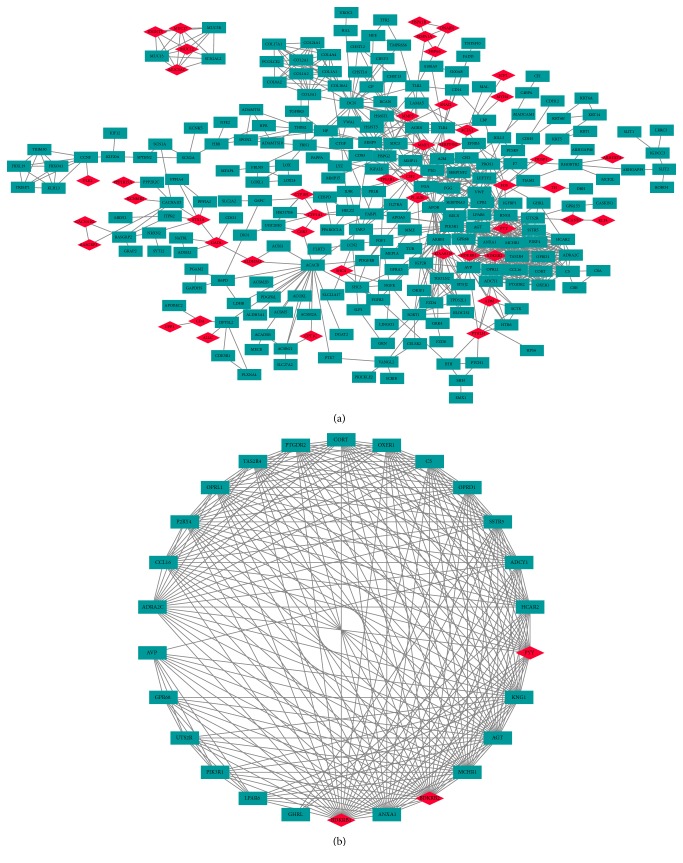
PPI network and the most significant module of DEGs. (a) The PPI network of DEGs was constructed using Cytoscape. (b) The most significant module was obtained from PPI network with 26 nodes and 234 edges. Upregulated genes are marked in light red; downregulated genes are marked in light blue.

To analyze the functional contributions of the DEGs, protein–protein interaction (PPI) network analysis was performed using STRING and Cytoscape. The false discovery rate (FDR) for each p-value was calculated. In general, the terms with FDR values < 0.01 were considered significantly enriched. The PPI network and the most significant module of the DEGs were determined (Figures 4(a) and 4(b)). Functional and pathway enrichment analyses of the genes involved in the module were performed using DAVID. Results showed that the genes in the most significant module were predominantly enriched for terms associated with inflammatory response, neuropeptide signaling pathway, and positive regulation of cytosolic calcium ion concentration (Table 3).

### 3.6. Hub Gene Analysis

**Figure 5:**
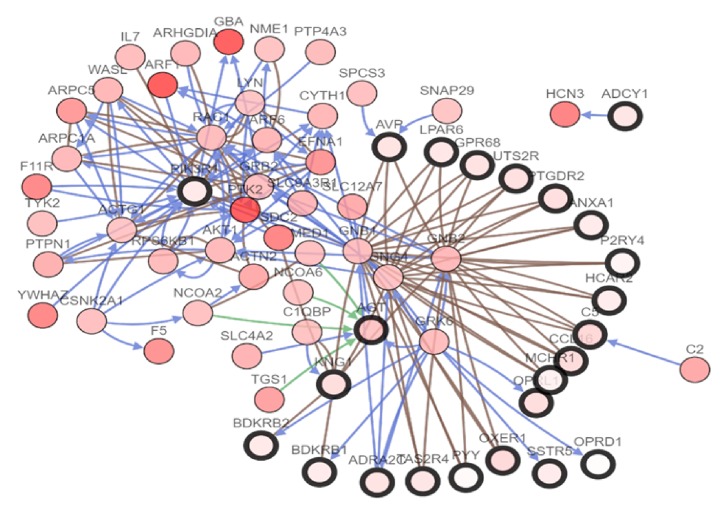
Interaction network analysis of the hub genes. Hub genes and their coexpression genes were analyzed using cBioPortal. Nodes with bold black outline represent hub genes. Nodes with thin black outline represent the coexpression genes.

**Figure 6:**
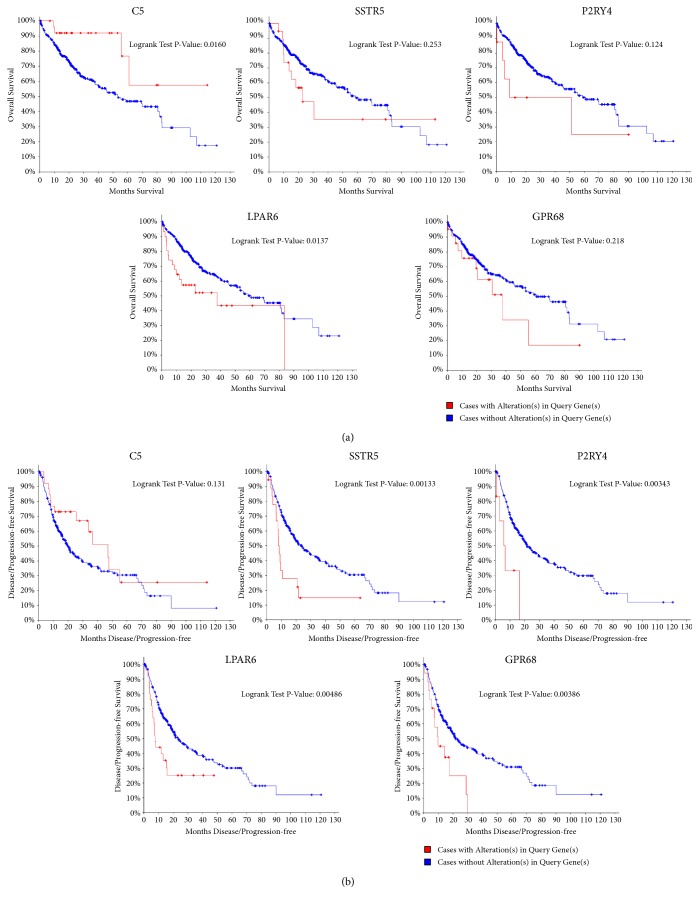
(a) Overall survival and (b) disease-free survival analyses of hub genes were performed using cBioPortal online platform. P<0.05 was considered statistically significant.

**Figure 7:**
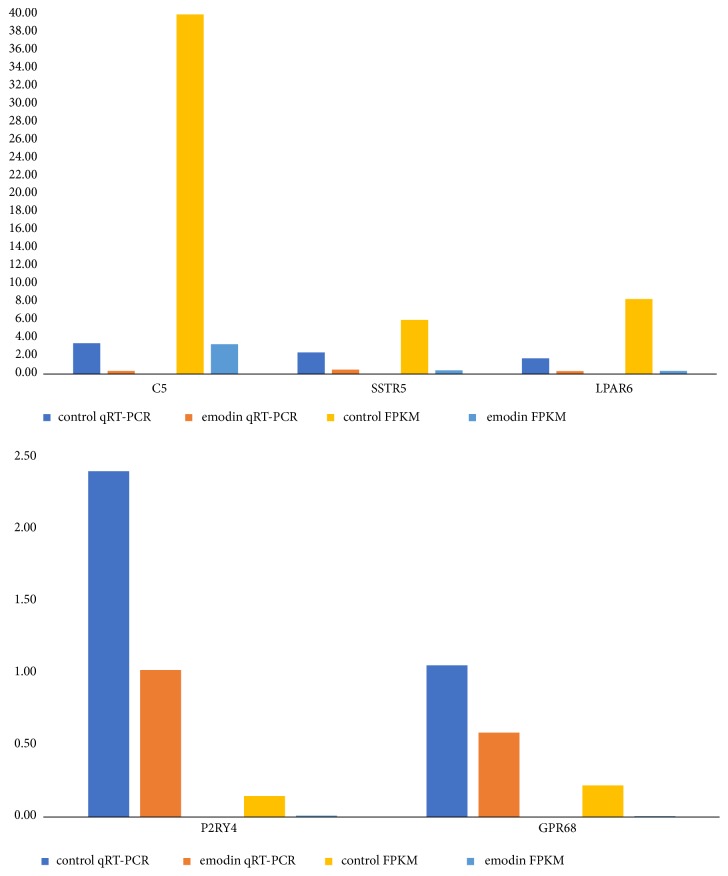
qRT-PCR validation of 5 differentially expressed hub DEGs. Comparison of fold change of LPAR6, C5, SSTR5, GPR68, and P2RY4 between RNA seq and qRT-PCR results.

A total of 25 genes were identified as hub genes with degree ≥10. The 25 most significant genes showing significant interaction were OPRD1, AVP, BDKRB2, TAS2R4, KNG1, BDKRB1, AGT, PTGDR2, LPAR6, C5, OPRL1, ADRA2C, CCL16, OXER1, CORT, SSTR5, PYY, MCHR1, UTS2R, ANXA1, ADCY1, GPR68, PIK3R1, P2RY4, and HCAR2. The functional roles of these hub genes are shown in Table 4. Coexpression of these hub genes was determined using cBioPortal (Figure 5). To further analyze these hub genes, overall survival and disease-free survival analyses were performed using a Kaplan-Meier curve. HCC patients with C5 and LPAR6 alterations showed low overall survival (Figure 6(a)), and HCC patients with SSTR5, P2RY4, LPAR6, and GPR68 alterations showed low disease-free survival (Figure 6(b)). Results based on qRT-PCR revealed that the mRNA levels of LPAR6, C5, SSTR5, GPR68, and P2RY4 were significantly downregulated in HepG2 cells (Figure 7). These findings indicated that C5, SSTR5, P2RY4, LPAR6, and GPR68 play important roles in the molecular mechanisms involved in emodin therapy for HCC.

## 4. Discussion

Transcription is an important biological process that determines the proteome of the cells. In this study, the RNA profile of HepG2 cells was determined via RNA-seq to explore the functions of DEGs in cancer cells treated with emodin. A total of 859 DEGs were screened, including 712 downregulated genes and 147 upregulated genes in HepG2 cells treated with emodin. These DEGs were found to be associated with emodin-mediated inhibition of liver cancer proliferation. Functional and pathway enrichment analyses of the 859 DEGs were performed using DAVID. GO analysis results showed that the DEGs were mainly enriched in regulation of apoptotic process and extracellular matrix organization. KEGG pathway analysis revealed that the DEGs were mainly enriched in mitogen-activated protein kinase (MAPK) signaling pathway. MAPK is a family of protein kinases comprising the p38 MAPKs, the c-jun N-terminal kinases (JNKs), and the extracellular regulated kinases (ERKs) [36]. MAPKs play crucial roles in regulating various cellular processes, such as cell proliferation. Our findings indicated that emodin-mediated inhibition of HCC cell proliferation is associated with the MAPK signaling pathway. The DEGs induced by emodin treatment were then carefully identified using a suite of sequence analysis software packages. Weighted gene coexpression network analysis has been successfully used to identify coexpression modules and intramodular hub genes based on DEG expression data. Analyzing the functional interactions between proteins can provide insights into the mechanisms underlying the effects of emodin in HCC. PPI networks were generated using Cytoscape and the most significant module in the PPI networks was identified using ClusterONE. The hub genes of the most significant module were then selected based on the cutoff degree ≥10. A total of 25 genes were identified as hub genes: OPRD1, AVP, BDKRB2, TAS2R4, KNG1, BDKRB1, AGT, PTGDR2, LPAR6, C5, OPRL1, ADRA2C, CCL16, OXER1, CORT, SSTR5, PYY, MCHR1, UTS2R, ANXA1, ADCY1, GPR68, PIK3R1, P2RY4, and HCAR2. The functional and pathway enrichment analyses of the 25 hub genes were performed using DAVID. GO analysis results showed that changes in BP of hub genes were significantly enriched in positive regulation of cytosolic calcium ion concentration, G-protein coupled receptor signaling pathway, adenylate cyclase-inhibiting G-protein coupled receptor signaling pathway, neuropeptide signaling pathway, and inflammatory response. Changes in CC of hub genes were mainly enriched in integral component of plasma membrane and plasma membrane. Changes in MF were mainly enriched in neuropeptide binding and G-protein coupled receptor activity. KEGG pathway analysis revealed that the hub genes were mainly enriched in neuroactive ligand-receptor interaction. To further analyze these hub genes, overall survival and disease-free survival analyses were performed using a Kaplan-Meier curve. Results showed that C5 and LPAR6 are associated with the overall survival of HCC patients. SSTR5, P2RY4, LPAR6, and GPR68 are related to the disease-free survival of HCC patients. These five genes are closely related to prognosis of HCC patients. Verification of the results by qRT-PCR showed that emodin can downregulate the mRNA levels of LPAR6, C5, SSTR5, GPR68, and P2RY4 in HepG2 cells (Figure 7). Therefore, C5, SSTR5, P2RY4, LPAR6, and GPR68 are likely to play important roles in the molecular mechanisms involved in emodin therapy for HCC. In summary, the five hub genes can reflect the molecular mechanisms of emodin therapy for HCC and could serve as targets for emodin therapy for HCC.

Complement activation is regulated to provide a permanent source of complement mediators that can maintain the inflammatory microenvironment that favors tumor growth [13, 37]. Complement component 5 (C5) is the fifth component of the complement and can be cleaved into C5a and C5b by C5-convertase. It plays a critical role in cell killing and inflammatory processes [38]. Many studies have reported that a variety of tumor cell lines can produce the complement activation product C5a. Furthermore, it has been reported that plasma C5a levels are elevated in liver cancer and other tissue-specific cancers [1, 6, 15]. He* et al.* showed that C5 levels were upregulated in AFP(-) HBV-related HCC and that C5 is potentially strongly associated with the progression of AFP(-) HBV-related HCC [17]. In addition, tumor inflammatory microenvironments were found to contain the complement-activating components C3, C4, C5, C1q, and MAC in many cancer models [39]. Somatostatin receptor type 5 (SSTR5) is a receptor that can lead to somatostatin-mediated inhibition of the release of hormones and secretory proteins [40]. A previous study reported that SSTR5 levels are upregulated in advanced-stage HCC [23]. SSTR5 can bind to somatostatin analogues, such as octreotide, which can help determine the antiproliferative efficacy of somatostatin analogues [2]. In addition, a positive correlation has been reported between SSTR5 expression and tumor size [8]. Lysophosphatidic acid receptor 6 (LPAR6) is a G protein-coupled receptor that can bind to lysophosphatidic acid [41]. One study reported that LPAR6 is essential for maintaining the tumorigenic properties of HCC cells; patient data and the experimental evidence supported the claim that LPAR6 promotes tumorigenicity and growth in HCC by activating the protooncogene Pim-3 [33]. Emodin can downregulate the expression of C5, SSTR5, and LPAR6. So far, no studies have examined the expression levels of P2Y purinoceptor 4 (P2RY4) and G-protein coupled receptor 68 (GPR68) in HCC. P2RY4, a G-protein coupled receptor, is responsive to uridine nucleotides [18] and plays an important role in transporting chloride in the epithelium of the jejunum [10]. The role of P2RY4 in apoptosis and cell proliferation, based on the effect of extracellular nucleotides, has been studied in HCT8 and Caco 2 cells [7]. In ovarian cancer, GPR68 is coupled to the PLC/Ca2+ pathway via the Gq/11 protein [42]. GPR68 is nearly undetectable in the healthy pancreas but is highly expressed in pancreatic ductal adenocarcinoma (PDAC) [34]. In human ovarian cancer cells, GPR68 has been shown to promote adhesion of cells to the extracellular matrix [35]. Among the five hub genes, C5, SSTR5, and LPAR6 were found to be involved in HCC (Table 5). These three genes could serve as the targets of emodin therapy for HCC (Table 5). However, few studies have investigated the relationships among P2RY4, GPR68, and liver cancer, and elucidating these relationships could be a direction for future research.

## 5. Conclusion

The present study attempted to identify DEGs that may be involved in the molecular mechanisms of emodin therapy for HCC. A total of 859 DEGs and 5 hub genes were identified and may be regarded as targets of emodin therapy for HCC. However, further studies are needed to elucidate the molecular mechanisms and biological function of these genes involved in emodin therapy for HCC.

## Figures and Tables

**Table 1:** General Statistics of Reads Alignment Process.

Sample	Total Raw Reads (M)	Total Clean Reads (M)	Total Mapping (%)
Emodin1	21.81	21.65	92.18
Emodin2	21.81	21.61	92.31
Emodin3	21.84	21.68	92.28
Control1	21.94	21.86	94.59
Control2	21.94	21.86	94.66
Control3	21.94	21.87	94.59

**Table 2:** GO and KEGG pathway enrichment analysis of DEGs in HepG2 cells treated with emodin. (Top 5) P<0.05 was considered statistically significant.

Category	Term	Count	P-Value
Upregulated			
GOTERM_BP	GO:0070370~cellular heat acclimation	3	3.01E-04
	GO:0043065~positive regulation of apoptotic process	10	3.04E-04
	GO:0032496~response to lipopolysaccharide	7	0.0011565
	GO:0090084~negative regulation of inclusion body assembly	3	0.0021952
	GO:2001240~negative regulation of extrinsic apoptotic signaling pathway in absence of ligand	4	0.0023164
GOTERM_CC	GO:0005576~extracellular region	23	0.0032977
	GO:0030016~myofibril	3	0.0179025
	GO:0005615~extracellular space	18	0.0200543
GOTERM_MF	GO:0042623~ATPase activity, coupled	3	0.0031569
	GO:0004252~serine-type endopeptidase activity	7	0.0097873
	GO:0008236~serine-type peptidase activity	4	0.0102052
	GO:0004947~bradykinin receptor activity	2	0.0141671
	GO:0019904~protein domain specific binding	6	0.0164848
KEGG_PATHWAY	hsa04010:MAPK signaling pathway	10	2.68E-04
	hsa05323:Rheumatoid arthritis	5	0.0066631
	hsa04915:Estrogen signaling pathway	5	0.0100336
	hsa05134:Legionellosis	4	0.010905
	hsa00980:Metabolism of xenobiotics by cytochrome P450	4	0.0252724
Downregulated			
GOTERM_BP	GO:0010951~negative regulation of endopeptidase activity	23	3.10E-10
	GO:0030198~extracellular matrix organization	25	1.58E-07
	GO:0007156~homophilic cell adhesion via plasma membrane adhesion molecules	21	9.99E-07
	GO:0042060~wound healing	14	5.01E-06
	GO:0042730~fibrinolysis	8	5.57E-06
GOTERM_CC	GO:0005615~extracellular space	114	8.37E-18
	GO:0005576~extracellular region	124	5.51E-16
	GO:0005578~proteinaceous extracellular matrix	35	1.88E-10
	GO:0016021~integral component of membrane	261	2.10E-10
	GO:0005887~integral component of plasma membrane	96	2.35E-09
GOTERM_MF	GO:0005509~calcium ion binding	63	8.97E-11
	GO:0004867~serine-type endopeptidase inhibitor activity	20	1.22E-09
	GO:0005201~extracellular matrix structural constituent	12	2.27E-05
	GO:0005198~structural molecule activity	23	7.88E-05
	GO:0001530~lipopolysaccharide binding	7	9.18E-05
KEGG_PATHWAY	hsa04610:Complement and coagulation cascades	18	2.02E-10
	hsa05146:Amoebiasis	16	5.35E-06
	hsa04512:ECM-receptor interaction	13	6.15E-05
	hsa04974:Protein digestion and absorption	13	6.90E-05
	hsa04611:Platelet activation	14	8.04E-04

**Table 3:** GO and KEGG pathway enrichment analysis of DEGs in the module. FDR<0.05 was considered statistically significant.

Category	Term	Count	FDR
GOTERM_BP	GO:0007204~positive regulation of cytosolic calcium ion concentration	10	2.54E-10
	GO:0007186~G-protein coupled receptor signaling pathway	15	5.11E-09
	GO:0007193~adenylate cyclase-inhibiting G-protein coupled receptor signaling pathway	6	9.72E-06
	GO:0007218~neuropeptide signaling pathway	6	4.76E-04
	GO:0006954~inflammatory response	7	0.0216325
GOTERM_CC	GO:0005887~integral component of plasma membrane	13	9.27E-05
	GO:0005886~plasma membrane	19	2.11E-04
GOTERM_MF	GO:0042923~neuropeptide binding	4	0.0035638
	GO:0004930~G-protein coupled receptor activity	8	0.0619499
KEGG_PATHWAY	hsa04080:Neuroactive ligand-receptor interaction	10	1.78E-05

GO, gene ontology; KEGG, Kyoto Encyclopedia of Genes and Genomes; DEGs, differentially expressed genes; FDR, false discovery rate

**Table 4:** Function roles of 25 hub genes with degrees ≥10.

No.	Gene symbol	Full name	Function
1	OPRD1	Delta-type opioid receptor	Play a role in the perception of pain and in opiate-mediated analgesia and developing analgesic tolerance to morphine.
2	AVP	Vasopressin-neurophysin 2-copeptin	It has a direct antidiuretic action on the kidney; it also causes vasoconstriction of the peripheral vessels. Acts by binding to vasopressin receptors (V1bR/AVPR1B, V1aR/AVPR1A, and V2R/AVPR2)
3	BDKRB2	B2 bradykinin receptor	Associated with G proteins that activate a phosphatidylinositol-calcium second messenger system.
4	TAS2R4	Taste receptor type 2 member 4	Encode a 7-transmembrane receptor protein, functioning as a bitter taste receptor.
5	KNG1	Kininogen-1	A mediator of inflammation and causes increase in vascular permeability.
6	BDKRB1	B1 bradykinin receptor	This is a receptor for bradykinin. Could be a factor in chronic pain and inflammation.
7	AGT	Angiotensinogen	Essential component of the renin-angiotensin system, a potent regulator of blood pressure, body fluid and electrolyte homeostasis.
8	PTGDR2	Prostaglandin D2 receptor 2	PI3K signaling is implicated in mediating PTGDR2 effects.
9	LPAR6	Lysophosphatidic acid receptor 6	Bind to oleoyl-L-alpha-lysophosphatidic acid (LPA). Intracellular cAMP is involved in the receptor activation.
10	C5	Complement C5	A mediator of local inflammatory process. Binding to the receptor C5AR1 induces a variety of responses including intracellular calcium release, contraction of smooth muscle, increased vascular permeability, and histamine release from mast cells and basophilic leukocytes
11	OPRL1	Nociceptin receptor	Play a role in the regulation of locomotor activity by the neuropeptide nociceptin.
12	ADRA2C	Alpha-2C adrenergic receptor	Alpha-2 adrenergic receptors mediate the catecholamine-induced inhibition of adenylate cyclase through the action of G proteins.
13	CCL16	C-C motif chemokine 16	Shows potent myelosuppressive activity and suppresses proliferation of myeloid progenitor cells.
14	OXER1	Oxoeicosanoid receptor 1	Receptor for eicosanoids and polyunsaturated fatty acids
15	CORT	Cortistatin	Bind to all human somatostatin receptor (SSTR) subtypes. It also inhibits cAMP production induced by forskolin through SSTRs.
16	SSTR5	Somatostatin receptor type 5	Increases cell growth inhibition activity of SSTR2 following heterodimerization
17	PYY	Peptide YY	This gut peptide inhibits exocrine pancreatic secretion, has a vasoconstrictory action, and inhibits jejunal and colonic mobility.
18	MCHR1	Melanin-concentrating hormone receptor 1	Receptor for melanin-concentrating hormone, coupled to both G proteins that inhibit adenylyl cyclase and G proteins that activate phosphoinositide hydrolysis.
19	UTS2R	Urotensin-2 receptor	High affinity receptor for urotensin-2 and urotensin-2B.
20	ANXA1	Annexin A1	Play important roles in the innate immune response as effector of glucocorticoid-mediated responses and regulator of the inflammatory process. Has anti-inflammatory activity
21	ADCY1	Adenylate cyclase type 1	Catalyzes the formation of the signaling molecule cAMP in response to G-protein signaling. Mediates responses to increased cellular Ca2+/calmodulin levels
22	GPR68	Ovarian cancer G-protein coupled receptor 1	Proton-sensing receptor involved in pH homeostasis.
23	PIK3R1	Phosphatidylinositol 3-kinase regulatory subunit alpha	Modulates the cellular response to ER stress during metabolic overloading in the liver and hence plays a role in glucose tolerance improvement
24	P2RY4	P2Y purinoceptor 4	Receptor for UTP and UDP coupled to G-proteins that activate a phosphatidylinositol-calcium second messenger system
25	HCAR2	Hydroxycarboxylic acid receptor 2	Receptor activation by nicotinic acid results in reduced cAMP levels which may affect activity of cAMP-dependent protein kinase A and phosphorylation of target proteins, leading to neutrophil apoptosis.

**Table 5:** Function roles of 5 genes in cancer.

Gene	Expression in cancer	Function in cancer	Ref
C5	Upregulated in HCC	Highly associated with the progression of AFP(-) HBV-related HCC.	[17]
SSTR5	Upregulated in HCC	Regulate intracellular signaling pathways, such as MAPK pathways; constitute a molecular basis for the treatment of HCC with somatostatin analogues.	[2, 32]
LPAR6	Upregulated in HCC	Maintain the proliferation capacity and the tumorigenic phenotype of HCC through the transcriptional activation of protooncogene Pim-3.	[33]
P2RY4	Upregulated in colonic cancer	The function is based on the effect of extracellular nucleotides on apoptosis or cell proliferation in HCT8 and Caco-2 cells.	[7]
GPR68	Upregulated in PDAC and ovarian cancer	Stimulates PDAC proliferation; inhibits ovarian cancer cell proliferation and migration, but enhances the cell adhesion to the extracellular matrix	[34, 35]

## Data Availability

The data used to support the findings of this study are included within the article.

## Abbreviations

HCC: Hepatocellular carcinoma
RNA-seq: RNA sequencing
DEGs: Differentially expressed genes
BP: Biological processes
CC: Cell component
MF: Molecular function
GO: Gene ontology
KEGG: Kyoto Encyclopedia of Genes and Genomes
DMSO: Dimethylsulfoxide
qRT-PCR: Quantitative real-time PCR
IC50: Inhibitory concentrations of 50%
MAPK: Mitogen-activated protein kinase
JNKs: c-jun N-terminal kinases
ERKs: Extracellular regulated kinases
C5: Component 5
SSTR5: Somatostatin receptor type 5
LPAR6: Lysophosphatidic acid receptor 6
P2RY4: P2Y purinoceptor 4
GPR68: Ovarian cancer G-protein coupled receptor 1
PDAC: Pancreatic ductal adenocarcinoma.
